# Notes and News

**Published:** 1903-09-19

**Authors:** 


### Notes and News.

The late Mr. Peter Brocklehurst, of Macclesfield, has bequeathed £5,000 to the Macclesfield Infirmary.

A case of plague has been reported from Yokohama, and the disease has also appeared at Inkau and Niu-Chwang.

Sir Victor Horsley will deliver the opening address of the Medical Faculty of the University of Birmingham on October 15th.

Mr. Astor has contributed the munificent sum of £20,000 to the Cancer Research Fund of the Royal Colleges of Physicians and Surgeons.

At the next meeting of the Society for the Study of Inebriety, on Tuesday, October 13th, at 8 p.m., in the Rooms of the Medical Society of London, 11 Chandos Street, Cavendish Square, London, W., Sir William Collins, M.D, F.R.C.S., D.L., L.C.C., will open a discussion on "The Institutional Treatment of Inebriety."

The late Miss M. F. Hasker, of St. Leonards, has bequeathed £1,000 to the Cancer Hospital, Brompton, £500 each to St. Mary's Hospital, Charing Cross, King's College and the London Hospitals, and to the Royal Hospital for Incurables, Putney, the Chelsea Hospital for Women, the Samaritan Free Hospital for Women and Children, the Hospital for Sick Children, Great Ormond Street, and the Hastings Free Dispensary, and the Home for Convalescent Children at West Hill, St. Leonards.

The subject of the eyesight of school children was discussed at the meeting of the British Association. The sub-committee on this subject reported that the principal cause of defects were defective and flickering light, faulty positions in regard to light during study, bad type and writing, and influences liable to affect the general health of the children. The sub-committee made recommendations to remedy these defects within the schools, and advocated the appointment of women inspectors of schools.

A serious outbreak of typhoid fever is reported from the Merthyr district. The contamination has been traced to cockles purchased in Merthyr market.

The Railway Commissioners in New South Wales have issued a regulation to the effect that spitting in railway carriages, smoking compartments not excluded, shall be punishable by a fine not exceeding £2.

The Washington authorities have put forward a proposal to place medical officers of health at European ports to inspect intending emigrants to America, with the view of preventing diseased persons from taking a needless voyage.

The authorities at Marseilles report that there is no spread of the plague and that every precaution has been taken, and affirm that there is no cause for alarm. Nevertheless, the Sanitary Council of Constantinople is imposing a five days' quarantine upon vessels coming from Marseilles.

It seems very unnecessary and a pity also that so much curiosity should have arisen over the exact cause of death of one so aged as the late Pope. The differences of medical men on the subject continue to be a question of public inquiry and discussion, although there can be no possible advantage in the inquiry to anyone.

The Prussian Ministry of Education appears to be thoroughly alive to the importance of proper school hygiene, and district medical officers receive special instructions to be watchful in these matters. They are recommending that new school buildings should be supplied with baths for the gratuitous use of the scholars.

Operations in connection with the new King's Sanatorium at Midhurst were commenced on 15th inst., when a large gang of men started digging the foundations, which have been undertaken by Messrs. Longley, of Horsham. The various portions of the work will be let to different contractors. Sir John Aird has secured the contract for the water supply and road-making, but the orders for the superstructure have not yet been placed.

A provincial sessional meeting of the Sanitary Institute will be held at the University of Birmingham, on Saturday, September 26th, at 11 a.m., when a discussion will take place on "Some Practical Considerations in connection with Modern Methods of treating Sewage." The discussion will be opened by Professor A. Bostock Hill, M.Sc., M.D., D.P.H., and J. E. Willcox, A.M.I.C.E., and general discussion is invited. The chair will be taken at 11 a.m. by Alfred Hill, M.D., F.R.S.E., F.I.C., F.C.S. Members will inspect the Birmingham Crematorium, Wallsall Road, near Perry Village, and visit the works of the Tame and Rea District Drainage Board at Tyburn, inspecting the sludge trenching operations on the way to Tyburn, where they will visit the site of quarter acre slag bacteria bed, Minworth Greaves, and newly laid-out irrigation lands in the neighbourhood. They will lastly inspect main effluent channels.

